# Retraction: Mechanism of action of novel piperazine containing a toxicant against human liver cancer cells

**DOI:** 10.7717/peerj.1588/retraction

**Published:** 2016-06-26

**Authors:** PeerJ Editorial Office

Retraction: Samie N, Muniandy S, Kanthimathi M, Haerian BS. (2016) "Mechanism of action of novel piperazine containing a toxicant against human liver cancer cells". PeerJ 4:e1588 https://doi.org/10.7717/peerj.1588

An investigation by PeerJ has determined that several of the images in the publication cited above have been inappropriately manipulated (for example Figure 7, Figure 12, Figure 13); that some of the images have been inappropriately re-used either in, or from, other publications (for example, versions of Figures 2 through 8 have all appeared in different publications suggesting to represent different cells, cell lines, or conditions (Samie et al., 2016a; Samie et al., 2016b)); and that large sections of the text have appeared in other publications (Samie et al., 2016a; Samie et al., 2016b; Muszalska et al., 2005).

These issues are currently being investigated by an ethics committee at the Faculty of Medicine, University of Malaya.

As a result, this article is Retracted.

This Retraction updates an Expression of Concern which was issued on June 10th 2016.
